# Dinner with Bayes: On the revision of risk beliefs

**DOI:** 10.1007/s11166-018-9294-2

**Published:** 2018-12-22

**Authors:** Christoph M. Rheinberger, James K. Hammitt

**Affiliations:** 1 European Chemicals Agency, Helsinki, Finland; 2 Center for Risk Analysis, Harvard University, Boston, MA USA; 3 Toulouse School of Economics, University of Toulouse, Toulouse, France

**Keywords:** Beliefs, Risk perception, Bayesian updating, Precautionary behavior, I12, I18, D80

## Abstract

We study how people form and revise health risk beliefs based on food safety information. In an online experiment, subjects stated their perceived risk of contracting a foodborne illness before and after receiving information about the population average risk and the eating habits of the average consumer. Precautionary effort in handling and preparing food reduced prior risk beliefs, but did not affect the belief revision process. About one quarter of subjects either fully ignored the information provided or revised their beliefs inconsistently with the Bayesian learning hypothesis. We find several factors related to the subjects’ numerical skills that explain information refusal and inconsistent belief revisions and discuss them in the context of health risks.

## Introduction

People often respond to public health policies in ways that are inconsistent with economic theory. They overreact to some risks while they ignore others (Slovic et al. 2000); they are reluctant to give up unhealthy behaviors though they know it would be better for them (O’Donoghue and Rabin 2001); and they take healthy behaviors as an excuse for indulging in unhealthy ones such as eating more when foods are low in calories (Wisdom et al. 2010) or smoking cigarettes down to the nub while trying to quit (Adda and Cornaglia 2006).

Reasons for these obvious deviations from the rational consumer model are manifold and include cognitive and attentional limitations, emotional arousal, various forms of procrastination, and difficulties in processing probabilistic information (McFadden 2006). In this paper, we study how consumers perceive an everyday health risk—contracting a foodborne illness—before and after the provision of relevant information. Since risk perception is a crucial link in the causal chain between consumer information and behavioral responses, understanding better how consumers form their beliefs and how they adjust them to new information is of considerable interest to policymakers (Magat and Viscusi 1992; Viscusi 1998). In the context of food safety, the interest is fueled by its implications for the evaluation of existing policies and by regulatory needs to accurately predict behavioral responses to new consumer information and awareness campaigns. Success or failure of such campaigns matters because each year foodborne pathogens cause billions of episodes of illness worldwide; in the U.S. alone, the annual welfare cost of foodborne illness is estimated to exceed $50 billion (Scharff 2012).

Two questions emerge. Do public information programs affect health risk perceptions? And if so, do they alter consumer behavior in the predicted manner? Answers to these questions require a better understanding of the processing of risk-related information. Indeed, the uptake of information plays a key role in studies of risk perception. Both economists and psychologists have long recognized that people make a number of common mistakes when they update risk beliefs with newly available information: small risks tend to be overestimated, while large ones tend to be underestimated (Kahneman 2003); risks are assessed based on emotions rather than cognitive evaluations (Loewenstein et al. 2001; Slovic et al. 2004); and more attention is given to bad news than to good news (Viscusi 1997).^1^

Over the past 30 years, a substantial number of empirical studies have addressed the impact of information on subjective risk beliefs. Here, we summarize the most important insights gained.

In a landmark study, Viscusi and O’Connor (1984) elicited chemical workers’ perception of job hazards based on warning labels and found that most workers displayed the capacity to consistently update their probabilistic beliefs with new information. In subsequent work, Viscusi (1997) studied location decisions in the presence of ambiguous information about air pollution and discovered that in contexts with multiple and conflicting sources of information respondents place disproportionate weight on alarmist information. Smith and Johnson (1988) studied the effect of public information programs on homeowners’ attitudes toward the health risk associated with radon exposure. Their results support a modified form of a Bayesian learning model for best describing individuals’ response to information about the risk of radon. Dickie and collaborators explored public perceptions of skin cancer (1996, 2007) and leukemia (Gerking et al. 2014) and consistently found respondents accounting for personal risk factors (e.g., complexion and sunlight exposure history) when assessing their own risk. They also observed that less knowledgeable and more concerned individuals demonstrated a greater propensity to use information provided.

While previous studies concluded that most people revise their risk beliefs in a manner broadly consistent with Bayesian inference, they largely ignored the endogenous nature of health risks with people often having private information about their health and taking precautionary measures to reduce the likelihood or severity of bad health outcomes. Private information and precautionary measures are typically unobserved in observational studies, but may systematically affect both perceptions and actions. For example, consumers choose the quality, storage place, and preparation of their foods, thereby affecting the likelihood of contracting a foodborne illness (Shogren and Stamland 2007). It might thus be perfectly rational for a consumer to believe her risk differs from the population average risk even if she is otherwise similar to the average consumer. Such an individualization of risk information may, however, be a major source of error because many information structures generate correlated rather than mutually independent signals. As Enke and Zimmermann (2018) demonstrate, many people fail to realize these dependencies and overshoot when forming or revising their beliefs.

This paper presents a belief-elicitation protocol that permits capturing the impact of precautionary behaviors and other idiosyncratic factors affecting both the formation and the revision of risk beliefs. In what is essentially a panel structure, a representative sample of French consumers stated their perceived chance of contracting a foodborne illness from eating fish. We first elicited subjects’ risk beliefs without providing any specific information. We repeated the elicitation after informing subjects about the average consumer’s fish consumption, the corresponding population average risk, and the prevalence of various risky and risk-averting behaviors. The chained elicitation procedure allows us to explore subjects’ responses to risk-related information, heterogeneity in the revision of risk beliefs, and deviations from the Bayesian rationality assumption that underlies the design of most—if not all—consumer information campaigns.

In a nutshell, we find that there is heterogeneity in belief revision but the majority of subjects updated their beliefs consistently with the Bayesian learning hypothesis. These subjects responded to information about the population average risk by reducing their prior beliefs if these were above the population average risk and by increasing their prior beliefs if they were below the population average risk. Precautionary effort in handling and preparing food reduced prior risk beliefs, but did not affect the belief updating process. This finding underpins the importance of controlling for confounding factors in understanding how individuals form and revise their risk beliefs and has implications for predicting the effectiveness of health and consumption advisories. For example, many respondents seem able to draw reasonable inferences from their precautionary behavior about their own risk and to use information about average risk and precautionary behavior in the population to update their beliefs in a Bayesian fashion. However, it is less clear that they can combine these pieces of information in a coherent manner, leading many respondents to update their beliefs quite drastically. Others do not consistently update at all and may be better served by more direct messages about the risks of certain behaviors.

Using finite mixture models we decompose the heterogeneity in belief revision and find four distinct updating patterns: (1) subjects who aggressively adjust their beliefs toward the population average risk; (2) subjects who modestly revise their beliefs toward the population average risk; (3) subjects who ignore the new information altogether; and (4) subjects who update in a manner inconsistent with the information provided. The first two patterns are entirely consistent with the Bayesian updating hypothesis, the third pattern may or may not be consistent depending on the reason to ignore new information, and the fourth pattern is clearly inconsistent. The mixture modeling approach allows us to link the emerging patterns to personal characteristics. We find that older, less educated and less numerate subjects are more likely to adapt either strategy (3) or (4) when updating their risk beliefs. Both refusal of information and lack of numeracy are problematic from the regulator’s point of view as they are associated with violation of Bayesian updating and undermine the efficacy of public health policies that seek to change behavior by informing consumers.

Our paper makes several contributions. We analyze a rich data set which enables us to disentangle the effects of endogenous precautionary effort on the formation versus the revision of risk beliefs. Unlike the studies cited above, our experimental task elicited risk beliefs conditional on a future foodborne illness, which requires respondents to evaluate the relative probabilities of multiple causes of foodborne illness rather than the marginal probability of illness. We believe this makes the updating less onerous and more tractable for subjects. Another innovation is that we estimate beta regression models to account for the theoretical underpinnings of the Bayesian learning model (Viscusi 1979). Finally, we account not only for observable, but also for unobservable heterogeneity in belief updating.

The paper proceeds as follows. In Section 2, we operationalize the Bayesian learning model and derive a formal definition of rational updating that is conditional on the precautionary effort expended by the updater. Section 3 provides details of the belief-elicitation task and the sample characteristics. Section 4 summarizes our econometric approach (details are given in the Appendix). Section 5 presents the results of our study. In Section 6, we discuss the response to information at the individual and the aggregate level. Section 7 concludes.

## Bayesian learning model

Ample evidence from both experimental and observational studies suggests that people overestimate the likelihood of rare events although the same people might underrespond to rare events in decisions from experience (de Palma et al. 2014). Since the seminal paper by Lichtenstein et al. (1978), dozens of studies have shown that this observation specifically applies to the context of health risks, with people being either overly optimistic or pessimistic about their risk of dying or of developing a specific disease.^2^

Economic theory holds that accurate information about the nature of the risk and the means of precaution may help people to better align their beliefs to the actual risk. Yet in the real world people might—willingly or unwillingly—ignore information. Viscusi (1989) assumed that subjects do not treat the probabilities presented to them as fully informative and proposed a model in which individuals use probabilistic information in a Bayesian fashion to revise their risk beliefs. He argued that the Bayesian updating process is consistent with two possible interpretations: individuals might not have full confidence in the source of information, or they might treat any risk-related information as not perfectly applicable to their individual circumstances. Both interpretations allow people to discount new information within the updating process. This can be formalized in the most basic version of the Bayesian learning model:

1 $$ q_{i}=\frac{\gamma p_{i}+\xi s}{\gamma+\xi}=\gamma^{*}p_{i}+\xi^{*}s, $$

where *q*_*i*_ denotes individual *i*’s posterior risk belief, which is formed based on *i*’s prior risk belief *p*_*i*_ and the information about the population average risk *s* (all of which are probabilities); *γ* and *ξ* are the information contents associated with *p*_*i*_ and *s*, respectively; and *γ*^∗^ = *γ*/(*γ* + *ξ*) and *ξ*^∗^ = *ξ*/(*γ* + *ξ*) are the corresponding precision weights.^3^

Equation 1 assumes that individuals form their posterior belief as a weighted average of the belief they held prior to receiving the risk-related information and the inference drawn from this information. A limitation of this simple Bayesian learning model is that it treats the interpretation of new information as a black box. Smith and Johnson (1988) proposed a behavioral refinement of the basic model in which factors that affect the inference process might also affect people’s perception of the relative precision of their prior beliefs, of the information content, or both. It is likely that some of these factors also affect the formation of prior risk beliefs.

Based on Smith and Johnson’s insights, we extend the basic Bayesian learning model to explore heterogeneity in the response to risk-related information. In particular, we assume that people process new pieces of information and combine them with personal knowledge of exposure and precautionary behavior to form their posterior risk belief:

2 $$ q_{i}=\gamma^{*}(\boldsymbol{\mathbf{A}}_{i},\theta_{\mathbf{A}})p(\mathbf{B}_{i},\theta_{\mathbf{B}})+\xi^{*}(\mathbf{C}_{i},\theta_{\mathbf{C}})s(\triangle\mathbf{D}_{i},\theta_{\mathbf{D}}), $$

where *𝜃*_∙_ are parameter vectors. The precision weights *γ*^∗^ and *ξ*^∗^ are contingent upon factors (summarized in vectors ***A***_*i*_ and ***C***_*i*_) that influence individual perception of the relative precision of the prior and the information, respectively. The prior risk belief *p*_*i*_ is a function of personal factors (age, gender, education, etc.) collected in the multidimensional vector **B**_*i*_. Similarly, the inference that the subject draws from the received risk information about the population risk *s* depends on a number of behavioral factors (exposure, precautionary effort, risky behavior, etc.). Instead of directly including these factors, we measure subject *i*’s behavioral distance from the average consumer by the *j*-dimensional vector ${\Delta }\mathbf {D}_{i}=\mathbf {D}_{i}-\bar {\mathbf {D}}$ (with $\bar {\mathbf {D}}$ denoting a vector of sample means). This deviation is crucial for *i*’s interpretation of the population average risk *s*. If *i* believes herself to be more or less exposed than the average consumer, she is likely to use *s* and Δ**D**_*i*_ as reference points for adjusting her prior belief accordingly (Hogarth and Einhorn 1992).

We invoke the behavioral implications of equation (2) to define a rational response to risk-related information. Consider individual *i*’s response to information about the average consumer’s behavior and the corresponding population average risk *s*. Let *d*_*i*_ denote any aggregating function of the behavioral deviations Δ**D**_*i*_ from the average consumer so that *d*_*i*_ > 0 (*d*_*i*_ < 0) means the subject behaves in a more (less) risky way than the average consumer. Given the consumer’s observed prior risk belief *p*_*i*_ and behavioral distance *d*_*i*_ to the average consumer, consistent updating implies the following.

### **Definition 1** (Consistent belief updating)

A consistent response to risk-related information does not violate any of the following conditions: (i) if *d*_*i*_ = 0 and *p*_*i*_ = *s*, then *s* = *p*_*i*_ = *q*_*i*_; (ii) if *d*_*i*_ = 0 and *p*_*i*_ < *s*, then *s* ≥ *q*_*i*_ ≥ *p*_*i*_; (iii) if *d*_*i*_ = 0 and *p*_*i*_ > *s*, then *s* ≤ *q*_*i*_ ≤ *p*_*i*_; (iv) if *d*_*i*_ > 0 and *p*_*i*_ ≥ *s*, then *s* ≤ *q*_*i*_; (v) if *d*_*i*_ > 0 and *p*_*i*_ < *s*, then *p*_*i*_ ≤ *q*_*i*_; (vi) if *d*_*i*_ < 0 and *p*_*i*_ > *s*, then *p*_*i*_ ≥ *q*_*i*_; (vii) if *d*_*i*_ < 0 and *p*_*i*_ ≤ *s*, then *s* ≥ *q*_*i*_.

**Fig. 1:**
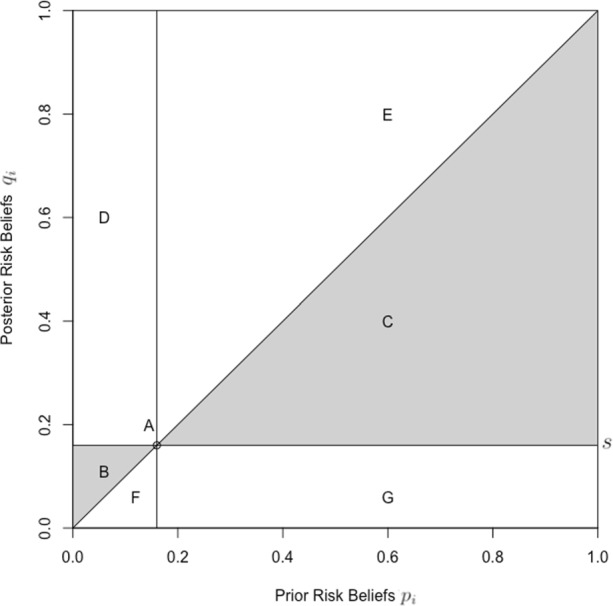
Landscape of possible belief revisions. *Notes*: Each of the consistency conditions of Definition 1 delimits a specific area: (i) $\sim $*A*; (ii) $\sim $*B*; (iii) $\sim $*C*; (iv) $\sim $*C*∪*E*; (v) $\sim $*B*∪*D*; (vi) $\sim $*C*∪*G*; (vii) $\sim $*B*∪*F*

Conditions (i)-(iii) provide a behavioral reformulation of equation (1) applying to those individuals who behave as does the average consumer and therefore do not have behavioral reasons to deviate from the announced population average risk *s*. Conditions (iv)-(vii) prescribe how an individual should respond when they engage in more or less risky behavior than the average consumer. Figure 1 maps out all the possible belief revisions. Compliance with the first three conditions corresponds to belief revisions in the gray-shaded area, whereas belief revisions outside the gray-shaded area are rational if, and only if, the subject differs from the average consumer in the manner prescribed by the latter four conditions.

Definition 1 classifies dynamically consistent belief revisions as rational behavior. That is, subjects who factor in both information about the average consumer and their personal behaviors may consistently update their beliefs, even if located outside the gray-shaded area. However, subjects may hold too optimistic or too pessimistic beliefs about the risks they face. Definition 2 classifies the observed belief revisions based on Spinnewijn’s (2013) concept of optimistic and pessimistic risk beliefs.

### **Definition 2** (Optimistic and pessimistic beliefs)

Subjects with risk beliefs located to the right (left) of the vertical line in Fig. 1 display baseline pessimism (optimism): before receiving the information, they perceive their risk to be larger (smaller) than the population average risk. Subjects with risk beliefs located above (below) the horizontal line in Fig. 1 display control pessimism (optimism): upon receiving the information, they believe their personal behavior raises (reduces) the risk above (below) the population average risk.

Definitions 1 and 2 will help us in the empirical part of the paper to identify inconsistent responses to risk-relevant information.

## Experimental design

We developed an experimental approach to measuring the revision of health risk beliefs in a representative sample of French consumers. Two premises guided the development: (1) people are not very good at making sense of small probabilities, but (2) they do fairly well in reporting expectations for specific states of the world as a percent chance (Manski 2004). The belief-elicitation task proceeded as follows. We first informed subjects about the annual number of cases of foodborne illness in France (about 250,000). Based on this information, they indicated on a semi-quantitative scale with eight categories (ranging from once per month to less than once in a lifetime) how frequently they expected to suffer a foodborne illness. That is, they gave us a crude estimate of their absolute risk of contracting a foodborne illness.

Next, we instructed subjects to assume they will suffer a foodborne illness sometime next year, and inquired how likely they thought it was (in terms of a percent chance) that the cause for their illness would be from eating fish.^4^ In other words, we elicited the conditional risk of a fishborne illness. The task was computer-based and subjects indicated their conditional risk estimates using a percent slider.

Subjects then received information about the fraction of cases of foodborne illness in France attributable to eating fish, behaviors that may reduce or increase the risk of contracting a foodborne illness, and the consumption and preparation habits of French fish consumers.^5^ We asked them to consider this information when revising their priors. This time the percent slider had additional marks indicating the subject’s prior and the number of cases attributable to fish consumption. Based on the information provided, subjects who knew the approximate population of France (about 66 million) could thus estimate the population annual average risk of foodborne illness (250,000/66 million $\thickapprox 4/1,000$) as well as the risk of illness from eating fish (16*%* × 250,000/66 million $\thickapprox 6/$10,000). With this estimate at hand, they could account for own behaviors when updating their prior risk belief. Any rational deviation from the population average conditional risk may thus be explained by the subject’s habits of preparation and consumption of fish and other foods, and their beliefs about the absolute risk of the different types of foods consumed.

We note that several non-Bayesian models of belief updating have been proposed. Perhaps the most prominent among them is support theory (Tversky and Koehler 1994; Rottenstreich and Tversky 1997), which presumes that subjective probability is not attached to events but to descriptions of events. Unpacking the description of a specific event into disjoint components is thought to increase its support. In our experimental setup unpacking is related to the new information. Provided with this information, subjects may draw inferences about the risk of contracting an illness from other foods than fish. Support theory predicts that subjects may reduce their prior belief about the conditional probability that their illness is due to fish, even if they assign the same unconditional probability to contracting a fishborne illness. Our definition of consistent updating permits subjects to draw such inferences—it requires only that belief revisions are dynamically consistent.

The belief-elicitation task was part of a large online survey which we administered after pretesting to a French consumer panel maintained by the survey firm CSA between July and September 2012. Respondents were limited to panel members aged 18 to 80. We obtained valid answers from 987 panel members. As the sample matches quotas for age, gender, region, and employment status, we take it to be representative of French consumers.^6^

The survey included multiple sections. To begin, respondents were asked about their age, occupation, work status, and frequency of fish consumption. Only respondents who reported eating fish at least two to three times per month continued to the main sections. These concerned: (1) health status and behaviors (e.g., smoking); (2) fish consumption (preferred species, quantities, storage and preparation methods); (3) concerns about health risks (e.g., cardiovascular disease, diabetes) and risks associated with fish consumption (e.g., pathogens, chemical pollutants, wild vs. farmed fish); (4) an experimental task presenting choices among three fish meals differing in species, form (e.g., filet vs. fish sticks, fresh vs. frozen), whether it had been tested for mercury or other contaminants, and price; (5) follow-up questions about the realism of the choices made in the experiment; (6) perceived efficacy of various precautions for reducing risk of becoming ill from consuming fish; (7) perceived risk that foodborne illness is due to fish consumption (the focus of this paper); (8) sociodemographic information about the respondent and household; and (9) a lottery task in which the respondent could choose one among a set of lotteries with different expected payoffs and risks. Table 1 summarizes our data set.

**Table 1:** Sample characteristics

Variable	Description	Obs.	Mean	St. Dev.	Min.	Max.
PRIOR	Prior risk belief (*p*)	987	0.31	0.25	0	0.99
POSTERIOR	Posterior risk belief (*q*)	987	0.23	0.20	0	0.93
BASELINE PESSIMISM	Prior risk belief larger than population average risk $(q>s)$	987	0.61	0.49	0	1
CONTROL PESSIMISM	Posterior risk belief larger than population average risk $(p>s)$	987	0.47	0.50	0	1
MALE	Gender is male	987	0.49	0.50	0	1
KIDS	Lives with children younger than 10 years	987	0.29	0.45	0	1
AGE	Subject’s age	987	43.54	13.51	18	80
INCOME	Monthly household income in €1,000	931	2.85	0.15	0.25	5
EDUCATION	Years of education	987	14.20	3.40	5	17
PREGNANCY	Pregnancy within the subject’s household	987	0.10	0.31	0	1
HEALTH STATUS	Self-assessed health status^a^	987	7.38	1.69	0	10
SAFETY CONCERNS	Subject has concerns over the safety of seafood	987	0.52	0.50	0	1
NUMERACY	Subject passed a simple numeracy test^b^	987	0.93	0.25	0	1
SUBJECTIVE RISK	Stated risk of foodborne illness per life-year	987	0.41	0.45	0	1
RAW FISH	Fraction of fish consumed raw (e.g. as sushi)^c^	987	0.96	1.75	0	10
WASH HANDS	No. of times hands are washed before meal preparation^c^	987	7.22	3.86	0	10
STORE FISH	No. of times fish is kept more than 3 days in the fridge^c^	987	0.83	2.14	0	10
PREPARE FISH	No. of times subject prepares fish well done^c^	987	5.97	4.07	0	10
EAT FISH	No. of times subject eats fish per week	987	1.97	1.12	0.58	5

*Notes*: $^{\text {a}}$Measured on a scale from 0 (‘very poor’) to 10 (‘very good’); $^{\text {b}}$the numeracy test presented subjects with two grids displaying risks of 5 in 10,000 and 10 in 10,000 and asked which one was larger; $^{\text {c}}$Measured on a scale from 0 (‘never’) to 10 (‘always’)

## Econometric approach

The belief elicitation task outlined above yields responses expressing prior and posterior beliefs about the risk of contracting a fishborne illness on the unit interval. The usual approach to the analysis of such responses is to transform the data to the real line (probit/logit regression) or to censor them (tobit regression). However, such models are prone to heteroskedasticity issues and the resulting coefficient estimates often lack interpretability (Kieschnick and McCullough 2003). In our empirical analysis, we therefore pursue a two staged approach. We first estimate (2) using a double-bounded tobit model with an intercept and the prior risk belief *p* as the only predictors.^7^ This has the advantage that the coefficient estimates are directly interpretable as $\beta _{0}=s\frac {\xi }{\gamma +\xi }$ and $\beta _{1}=\frac {\gamma }{\gamma +\xi }$. One can then back out the average weight of information relative to the prior belief, ${\Psi }=\frac {\xi }{\gamma }=\frac {1-\beta _{1}}{\beta _{1}}$, where higher values of Ψ imply more weight given to the information provided (Viscusi and O’Connor 1984).

Our second approach builds on the double-index beta regression model introduced by Ferrari and Cribari-Neto (2004) to account for heterogeneity in risk beliefs. We give a detailed description in the Appendix, but note here that the beta regression model is tailored to the analysis of probabilistic beliefs as it fits the first two moments, contingent on observables, of the beta distribution that best describes the empirical data. As an extension, we present a finite mixture of beta regressions to account for heterogeneity in belief updating that cannot be explained by observable factors. The goal of the mixture modeling is to estimate the full set of parameters for each of *k* = 1,2,…,*K* latent classes along with a membership function, so that the model predicts to which class each observation most likely belongs (McLachlan and Peel 2000).

## Results

We arrange the presentation of our results around the following three questions: Do people update their beliefs in a Bayesian manner? What factors account for heterogeneity in belief updating? And what factors drive inconsistent belief revisions?

### Observed belief updating

**Fig. 2:**
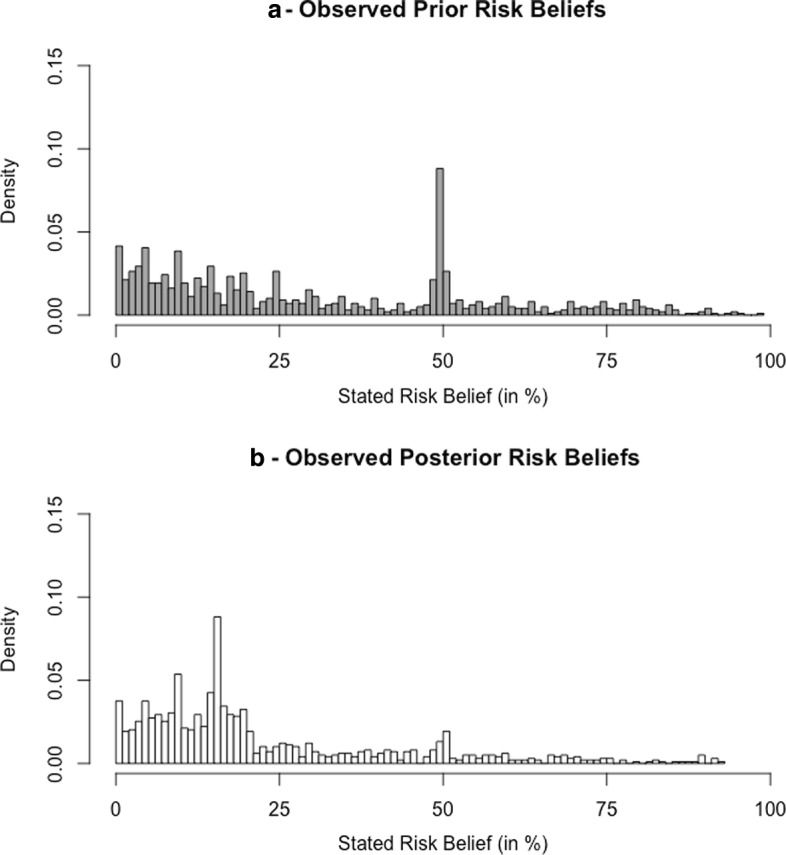
Histograms of observed beliefs

The histogram in panel a of Fig. 2 illustrates that, before receiving information about the average consumer’s risk, subjects maintained relatively pessimistic prior beliefs with both mean (*E*(*p*) = 31*%*) and median (*M*(*p*) = 25*%*) significantly larger than the population average risk (*s* = 16*%*). A significant spike at 50% suggests that some subjects had “no idea as to the answer” (Fischhoff and Bruine De Bruin 1999). The provision of risk-related information reduced the perceived risk significantly (*E*(*q*) = 23*%*, *M*(*q*) = 16*%*) and smoothed the spike (Fig. 2, panel b). The mean posterior subjective risk belief was still almost 8 percentage points higher than the population average risk *s*, suggesting that on the sample level subjects displayed control pessimism. The vast majority (817 out of the 987 subjects) revised their beliefs in response to the information provided, with 650 subjects updating within the gray-shaded area of Fig. 1. Among them, 87 subjects updated their beliefs to perfectly match the population average risk whereas 170 subjects dismissed the information altogether and retained their prior beliefs.

We report the results of the tobit model outlined in Section 4 in Table 2. Based on the coefficient estimates of the tobit model we derive a relative weight of about 0.8 given to the information, meaning subjects gave on average about 20% more weight to their prior than they gave to the information provided. This is significantly less than the workers in the Viscusi and O’Connor study (1984) gave to the chemical labeling information, but the result is consistent with the results obtained in the studies by Dickie and Gerking (1996, 2007) and Gerking et al. (2014) which found that respondents put more weight on their prior than they put on information when assessing their risk of developing different types of cancer.

**Table 2:** Relative informational weight: Results of tobit regression model

	Est.		Std. error
(*β*_0_) (Weight given to the risk information)	0.055	***	0.006
(*β*_1_) (Weight given to the prior risk belief)	0.549	***	0.024
(Ψ) (Relative informational weight)	0.821		
Observations	987		
Log-likelihood	− 460		
AIC	− 915		
BIC	− 900		

Notes: $^{***}P<0.01,{}^{**}P<0.05,{}^{*}P<0.1$

### Observed heterogeneity in posterior beliefs

It is widely accepted that personal characteristics and world views mediate the perception of risks (Slovic et al. 2000). The same factors may also affect how new information is processed when beliefs about the corresponding risks are updated. As risk beliefs are bounded to the unit interval, we next explore what drives heterogeneity in the updating process using the beta regression model described in the Appendix.

Table 3 reports on two distinct specifications. The main-effects-only model ignores that factors which affect the belief updating might also affect the formation of prior risk beliefs and/or the precautionary effort expended by the subject. In other words, the specification presumes—perhaps naively—that there is no interaction between the vectors ***A***_*i*_, ***B***_*i*_, ***C***_*i*_, and Δ***D***_*i*_. In contrast, the interaction-effects model includes two-way interactions between the prior risk belief and each of the precautionary behaviors, between the prior and each of the socioeconomic characteristics, and between each of the precautionary behaviors and each of the socioeconomic characteristics, as well as three-way interactions between each of the subject’s socioeconomic characteristics, each of the precautionary behaviors, and the prior risk belief.

**Table 3:** Observed updating: Results of beta regression models

	Main-effects-only model					Interaction-effects model				
	Est.		Std. error	Data range	1^st^Diff.	Est.		Std. error	Data range	1^st^diff.
(*μ*)_(Intercept)	–1.874	***	0.261			–2.111	***	0.466		
(*μ*)_PRIOR	3.222	***	0.126	[0.0; 1.0]	0.608	3.522	***	1.242	[0.0; 1.0]	0.595
(*μ*)_HEALTH STATUS	–0.004		0.018	[0.0; 10.0]	–0.007	0.031		0.024	[0.0; 10.0]	–0.023
(*μ*)_PREGNANCY	0.107		0.077	[0.0; 1.0]	0.017	0.067		0.158	[0.0; 1.0]	0.017
(*μ*)_MALE	–0.124	**	0.059	[0.0; 1.0]	–0.019	0.115		0.103	[0.0; 1.0]	–0.017
(*μ*)_KIDS	0.045		0.063	[0.0; 1.0]	0.007	–0.159		0.117	[0.0; 1.0]	0.005
(*μ*)_AGE	0.000		0.002	[18.0; 80.0]	0.003	–0.008	**	0.004	[18.0; 80.0]	0.011
(*μ*)_INCOME^b^	–0.022		0.029	[0.25; 5.0]	–0.016	0.008		0.052	[0.25; 5.0]	–0.018
(*μ*)_EDUCATION	–0.023	***	0.008	[4.0; 17.0]	–0.044	–0.028	*	0.014	[4.0; 17.0]	–0.047
(*μ*)_NUMERACY	–0.143		0.118	[0.0; 1.0]	–0.023	0.048		0.349	[0.0; 1.0]	0.007
(*μ*)_SUBJECTIVE RISK	0.154	***	0.057	[0.0; 1.0]	0.024	0.091		0.103	[0.0; 1.0]	0.023
(*μ*)_SAFETY CONCERNS	0.049		0.054	[0.0; 1.0]	0.008	0.199	**	0.092	[0.0; 1.0]	0.001
(*μ*)_EAT FISH^c^	0.010		0.023	[0.57; 5.0]	0.007	1.202	***	0.341	[0.57; 5.0]	0.028
(*μ*)_RAW FISH^c^	0.028		0.018	[0.0; 10.0]	0.046	–0.182		0.296	[0.0; 10.0]	0.067
(*μ*)_WASH HANDS^c^	–0.016	**	0.007	[0.0; 10.0]	–0.026	–0.046		0.117	[0.0; 10.0]	–0.031
(*μ*)_STORE FISH^c^	0.026	**	0.011	[0.0; 10.0]	0.043	0.159		0.195	[0.0; 10.0]	0.038
(*μ*)_PREPARE FISH^c^	–0.011		0.007	[0.0; 10.0]	–0.018	–0.055		0.122	[0.0; 10.0]	–0.023
(*ϕ*)_(Intercept)	2.528	***	0.107			2.688	***	0.083		
(*ϕ*)_BASELINE PESSIMISM	–0.888	***	0.141			–0.979	***	0.119		
(*ϕ*)_CONTROL PESSIMISM	–1.046	***	0.266			–1.299	***	0.251		
(*ϕ*)_PESSIMISM INTERACTION	1.501	***	0.294			1.696	***	0.275		
Interaction effects	excluded					included $(\chi ^{2}= 154.0,115$ Df)				
Observations	987					987				
Log-likelihood	–819					–896				
AIC	–1,595					–1,519				
BIC	–1,493					–854				

Notes: $^{***}P<0.01,{}^{**}P<0.05,{}^{*}P<0.1;$$^{\text {a}}$ mean-centered variables

In both specifications, we model the precision in belief updating as a function of attitudes toward baseline risk and risk controllability as we hypothesize that they affect the variability in observed updating behavior.

Since the interpretation of interaction effects in generalized linear models is complicated by the link function (Ai and Norton 2003), we discuss the major findings of the beta regression analysis in terms of change in the predicted outcome when one variable is set to its minimum and maximum value, respectively, while keeping the other variables fixed at their sample means (known as “first differences”). In the text we report, where meaningful, the average effect per unit obtained by dividing the first difference estimate by the corresponding value range.

Results of the main-effects-only model in Table 3 suggest that precautionary effort plays an important role in understanding the updating of subjective risk beliefs. In particular, subjects who take more (less) precaution than the average consumer as measured by the mean-centered variablesRAW FISH,WASH HANDS,STORE FISH,PREPARE FISH, andEAT FISH stated lower (higher) posterior risk beliefs. First differences indicate that heterogeneity in each of the recorded precautionary behaviors may account for differences in posterior risk beliefs of 0.7 to 4.6 percentage points.

Perceived vulnerability mattered less than expected. The only significant predictor is the SUBJECTIVE RISK of suffering a foodborne illness, which was associated with a 2.4 percentage point larger posterior risk belief among subjects who reported they bear a high risk. In agreement with the gender effect observed in previous risk perception studies (Slovic et al. 2000), MALE subjects had a 1.9 percentage point smaller posterior risk belief than female subjects. Posterior risk beliefs were negatively associated with EDUCATION, decreasing by about 0.3 percentage points per additional year of schooling. Notably, other indicators related to risk literacy such as the NUMERACY test and the AGE of the subject had no statistically significant association with the revised risk belief. Unsurprisingly, the modeled precision in belief updating is highest among subjects displaying neither BASELINE PESSIMISM nor CONTROL PESSIMISM.

Although the results of the main-effects-only model are consistent with what one would intuitively expect, the results of the interaction-effects model in Table 3 call for a more cautious interpretation. Once we include interactions between prior risk beliefs and the characteristics of the subject, none of the coefficients related to the precautionary behaviors—except EAT FISH—remains significant. Yet the corresponding first differences are comparable in size to those of the main-effects-only model, suggesting that precautionary behaviors affect the formation of the prior risk belief rather than the updating. We conclude that ignoring these interdependencies leads to overestimating the impact of precautionary behavior on the revision of beliefs.

Another reason to control for confounding effects is that behavioral drivers of belief updating may be masked by complex interactions between precaution, exposure and prior risk belief. Indeed, the results of the interaction-effects model suggest that subjects increased their posterior risk belief by 0.6 percentage points per additional fish meal (EAT FISH), meaning that subjects clearly responded to the quantitative part of the information provided. Even after controlling for the impact of educational attainment on the formation of the prior risk belief (insignificant coefficient not reported in Table 3), subjects stated almost 0.4 percentage points lower posterior beliefs per additional year of EDUCATION. This finding indicates that better educated subjects put more weight on the information if their prior risk belief was higher than the population average risk. Other indicators of numerical skills seem not to be associated with the updating of risk beliefs, however. Again, homogeneity in belief updating is greatest among subjects displaying neither BASELINE PESSIMISM nor CONTROL PESSIMISM, suggesting that pessimistic attitudes are associated with more heterogeneous belief revisions.

**Fig. 3:**
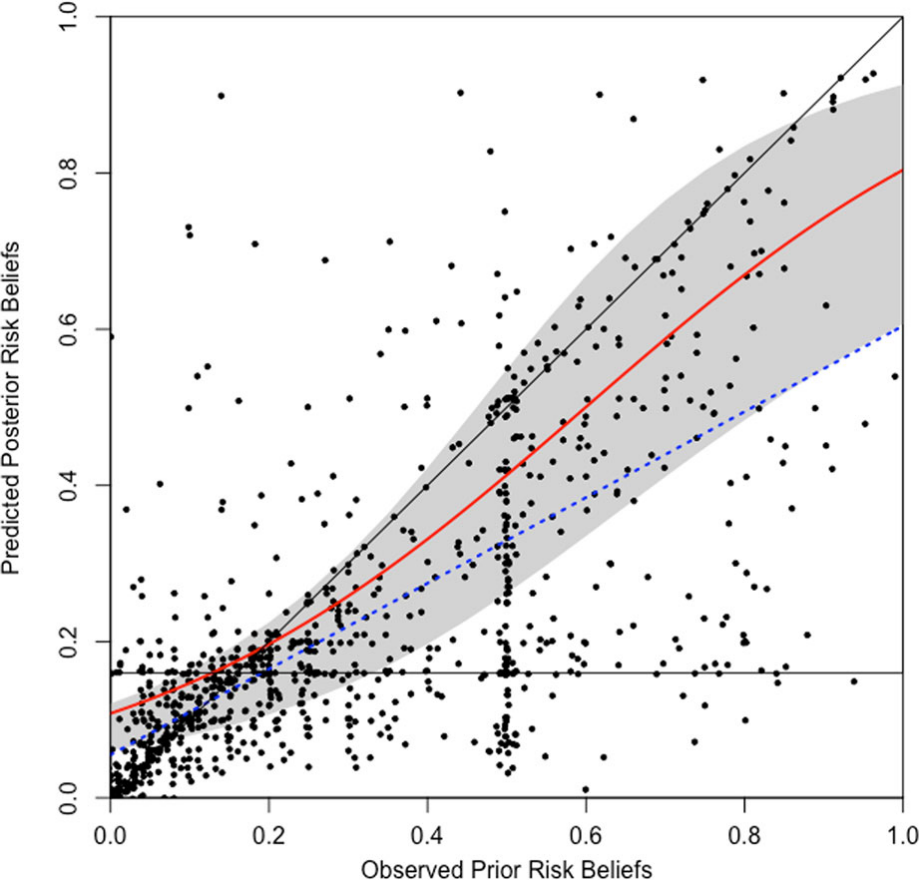
Predicted belief revisions. *Notes*: The dashed line indicates the prediction of the simple tobit model reported in Table 2; the continuous line indicates the counterfactual prediction of the interaction-effects model in Table 3 for the average subject; gray-shaded contours delineate counterfactual predictions for the most risky and most precautionary subjects, respectively (see the main text for explanation)

We use the coefficient estimates of the interaction-effects model reported in Table 3 to predict posterior beliefs assuming particularly risky and precautionary behaviors, respectively. The continuous line in Fig. 3 represents the predicted posterior risk from this model specification while the dashed line represents the posterior risk predicted from the tobit model reported in Table 2. Both predictions confirm that—consistent with the Bayesian learning model—the average subject adjusted their beliefs upward (downward) if their prior was smaller (larger) than the population average risk. The gray-shaded area delineates the range of predictions obtained by setting the independent variables at their sample minima and maxima. It suggests that only those subjects who take the least precautionary measures are expected to increase (reduce) their prior risk beliefs upon receiving the information if their priors were above (below) the population average risk.^8^

### Unobserved heterogeneity in posterior beliefs

So far, we have implicitly assumed that heterogeneity in posterior beliefs can be fully explained by observable characteristics of the subject. Yet there is good reason to believe that unobserved characteristics may affect the revision process so that the response to information provided varies from one group of subjects to another (Hogarth and Einhorn 1992). We model such latent classes of belief updaters using discrete mixtures of beta regressions (see the Appendix for details). Specifically, we assume for each latent class *k* that the belief updating function *μ*_*k*_ is a function of the prior risk belief as well as a number of predictors related to the precautionary effort of the subject (see Table 4). The membership function *π*_*k*_ includes EDUCATION, NUMERACY, AGE, and other socio-economic predictors as concomitant variables.^9^ As the identification of the latent classes is co-determined by these factors, the model includes a unique precision term *ϕ*_*k*_ for each class *k*.

**Table 4:** Unobserved updating: Results of beta regression mixture model with four latent classes

	Class 1: inconsistent updaters			Class 2: reluctant updaters			Class 3: aggressive updaters			Class 4: information refuseniks		
	Est.		Std. error	Est.		Std. error	Est.		Std. error	Est.		Std. error
(*μ*)_(Intercept)	–2.000	***	0.134	–2.520	***	0.112	–2.447	***	0.114	–2.491	***	0.050
(*μ*)_PRIOR	2.956	***	0.357	1.675	***	0.286	3.610	***	0.314	4.964	***	0.097
(*μ*)_EAT FISH^a^	–0.085		0.069	–0.001		0.042	–0.009		0.037	0.014		0.015
(*μ*)_RAW FISH^a^	0.057		0.040	–0.002		0.025	0.030		0.018	0.004		0.008
(*μ*)_WASH HANDS^a^	–0.030		0.020	–0.003		0.011	–0.013		0.013	0.002		0.004
(*μ*)_STORE FISH^a^	0.058		0.039	–0.018		0.021	0.006		0.019	0.002		0.006
(*μ*)_PREPARE FISH^a^	–0.002		0.019	–0.016		0.011	–0.018		0.015	0.002		0.005
(*ϕ*)_(Intercept)	1.240	***	0.135	3.354	***	0.244	3.718	***	0.566	5.854	***	0.298
(*π*)_(Intercept)	fixed to 0			–3.064		2.001	–0.453		1.560	–0.079		1.383
(*π*)_HEALTH STATUS	fixed to 0			0.077		0.137	0.158		0.110	–0.087		0.105
(*π*)_PREGNANCY	fixed to 0			–0.518		0.652	–0.046		0.658	–0.275		0.683
(*π*)_MALE	fixed to 0			0.448		0.443	0.285		0.424	0.006		0.408
(*π*)_KIDS	fixed to 0			–0.949	**	0.436	–0.623		0.443	–1.143	**	0.452
(*π*)_AGE	fixed to 0			–0.051	**	0.020	–0.056	***	0.018	–0.043	**	0.017
(*π*)_INCOME	fixed to 0			0.413	**	0.196	0.249		0.206	0.434	**	0.183
(*π*)_EDUCATION	fixed to 0			0.169	**	0.067	0.001		0.058	0.085		0.060
(*π*)_NUMERACY	fixed to 0			2.471	*	1.280	1.683	**	0.805	0.548		0.533
(*π*)_SUBJECTIVE RISK	fixed to 0			–0.553		0.478	0.848	*	0.452	0.434		0.455
(*π*)_SAFETY CONCERNS	fixed to 0			–0.720	*	0.393	–0.757	*	0.396	–0.531		0.389
Membership size^b^	154			330			296			207		
Observations	987											
Log-likelihood	–1,001											
AIC	–1,872											
BIC	–1,554											
ICL	-1,538											

Notes: $^{***}P<0.01,{}^{**}P<0.05,{}^{*}P<0.1;$$^{\text {a}}$ mean-centered; ^b^ membership size determined based on the number of subjects assigned with $\tau _{ik}^{(*)}=\max \tau _{iu}^{(*)} \forall u$ to class *k*

**Fig. 4:**
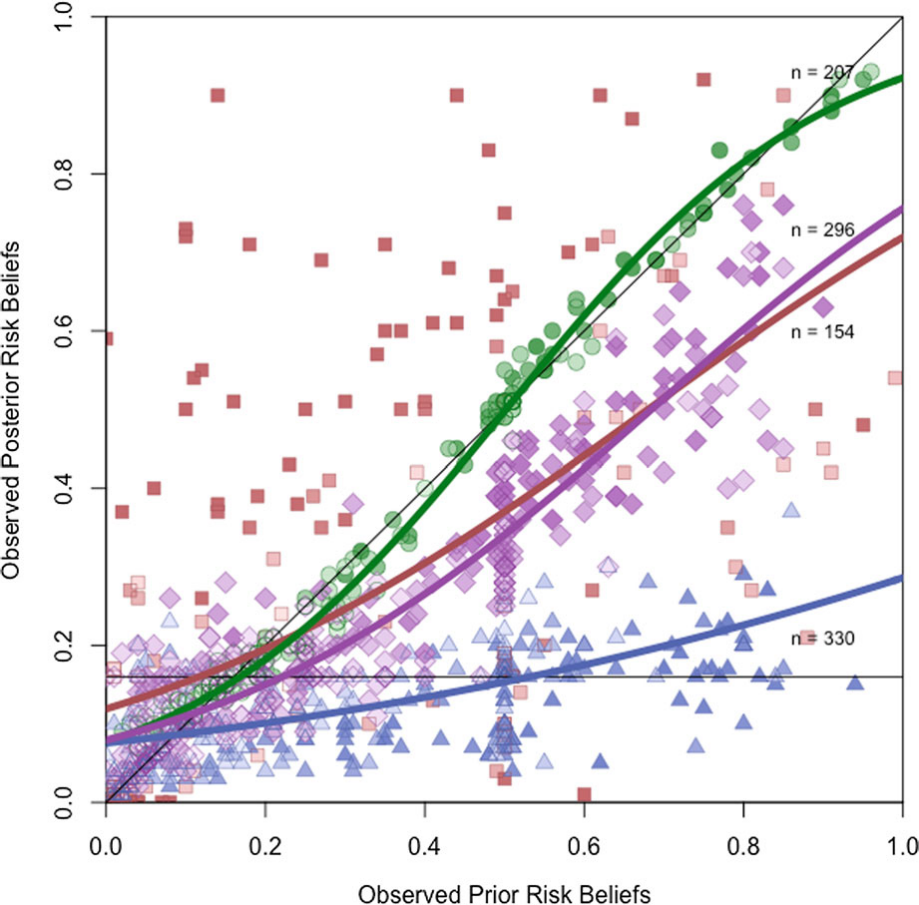
Latent patterns of belief updating. *Notes*: Shapes reflect class memberships: circles mark information refuseniks, diamonds mark reluctant updaters, squares mark inconsistent updaters, and triangles mark aggressive updaters; brighter colors indicate higher class membership probability at convergence; lines represent class-specific beta regression fits

We explored beta regression mixtures with up to five latent classes. Our preferred mixture model clusters the observed relationship between prior and posterior beliefs into four latent classes.^10^ Figure 4 shows the class-specific updating functions and the corresponding class membership probabilities at convergence, with brighter colors indicating a higher probability of belonging to class *k*. The classification is based on the highest class membership probability at convergence, i.e. subject *i* belongs to latent class *k* iff $\tau _{ik}^{(*)}=\max \tau _{iu}^{(*)} \forall u$ (where $\hat {\tau }_{ik}^{(*)}$ is subject *i*’s probability of belonging to class *k* at convergence). Each of the four classes represents a distinct pattern of belief revision. Circles represent 207 subjects who ignored the information provided and refused to substantially revise their risk beliefs. We refer to this class as *information refuseniks*. Triangles represent the class of *aggressive updaters*, comprising 330 subjects whose revised risk beliefs are close to the population average risk *s*. Diamonds represent the class of *reluctant updaters*, including 296 subjects who combined their prior risk beliefs and the information received in a manner consistent with the Bayesian learning hypothesis to form a new posterior risk belief. Lastly, squares represent 154 subjects whose belief revisions violate consistent updating as defined in Section 3 and to whom we therefore refer as*inconsistent updaters*.

One way of formally comparing the mixture model classification to Definition 1 is to operationalize the behavioral distance of the subject as the membership-weighted sum of deviations from the average consumer, i.e. $\hat {d}_{i}={\sum }_{k}\hat {\tau }_{ik}^{(*)}{\sum }_{j}\hat {\theta }_{jk}(\bar {D_{j}}-D_{ij})$ where $\hat {\tau }_{ik}^{(*)}$ is defined as above, and $\hat {\theta }_{jk}$ denotes the class-*k* specific coefficient on the mean-centered variable *j* as reported in Table 4. If the subject behaves like the average consumer, then $\hat {d}_{i}$ equals zero; it becomes positive (negative) if the subject takes more (less) risk. Comparing the resulting classification to the one predicted by the mixture model, we find an almost 80% overlap.

The results reported in Table 4 and displayed by Fig. 4 warrant further remarks. First, the classification obtained from the mixture model is far from crisp; i.e., for most subjects the maximum probability of membership in any class is much less than one. We find that $\hat {\tau }_{ik}^{(*)}\geq \frac {1}{2}$ holds for about 78% of the observations, $\hat {\tau }_{ik}^{(*)}\geq \frac {2}{3}$ holds for 47% of the observations, and $\hat {\tau }_{ik}^{(*)}\geq \frac {9}{10}$ holds for 18% of the observations. Such a noisy classification is not unusual in empirical applications of mixture models and reflects the inverse relationship between the number of latent classes and the discriminatory power of the mixture model.

Second, the coefficient estimates indicate that the precision in updating as measured by the class-specific precision parameter *ϕ*_*k*_ is up to five times smaller among inconsistent updaters than among members of the other classes. This highlights the randomness in the belief revision of inconsistent updaters. Aggressive and reluctant belief updaters display almost the same precision. In contrast, information refuseniks have a very high precision estimate. The posterior beliefs of this class are very close to their prior beliefs, whether low or high.

Third, the updating behaviors of the four classes are distinctly different. Consistent updaters increase (reduce) their posterior risk beliefs if their fish consumption is higher (lower) than average, with information refuseniks giving least weight and aggressive updaters giving most weight to their actual fish consumption. In contrast, inconsistent updaters who eat fish more (less) often than the average subject reduce (increase) their posterior risk belief in response to risk information. As a result, their belief revisions vary widely. This becomes even more evident if one compares the empirical distributions of belief revision of the four classes against each other. As Table 5 shows, inconsistent updaters are the only group in which one out of four subjects substantially raises their prior beliefs. On the other hand, a large fraction of inconsistent updaters aggressively reduced their priors, so that the mean and median of the empirical distribution of belief revision are close to zero. The same holds—almost by definition—for the group of information refuseniks, whereas aggressive and reluctant updaters lowered their prior beliefs by 19 and 7 percentage points on average.

**Table 5:** Group-specific empirical distributions of belief revision (posterior minus prior belief)

	Obs.	Min.	1st Qu.	Median	Mean	3rd Qu.	Max.
*Belief revisions*							
Aggressive updaters	330	–0.79	–0.34	–0.15	–0.19	0.00	0.16
Reluctant updaters	296	–0.37	–0.15	–0.07	–0.07	0.00	0.18
Information refuseniks	207	–0.07	–0.01	0.00	0.00	0.01	0.07
Inconsistent updaters	154	–0.67	–0.08	0.00	0.01	0.16	0.76

### Inconsistent belief updating

Estimates of the class membership function reported in Table 4 suggest that several concomitant variables influence the subject’s likelihood of belonging to a specific class *k* (the estimated average marginal effects for all concomitant variables in Table 4 are reported in the Appendix).^11^ Of particular interest is the probability of being classified as an inconsistent updater. This probability increases on average by about 0.5 percent with each year of AGE. Subjects who flunked the NUMERACY test have an 18 percent higher chance to be classified as an inconsistent updater, while those who had SAFETY CONCERNS about the fish they ate have a 7 percent higher chance. With each additional year of EDUCATION the likelihood of inconsistent updating decreases by a percentage point; similarly, it decreases by 3.5 percentage points for every additional €1,000 of monthly household INCOME.

The above results suggest that inconsistent updaters were either unable to cope with the complexity of the belief elicitation task, or failed to incorporate the new information into their existing beliefs. Which explanation is more accurate? To answer this question, we compare prior and posterior beliefs of inconsistent updaters against those of all other updaters. Table 6 suggests that inconsistent updaters held similar prior beliefs as the other subjects, but failed to incorporate new information in a consistent way as indicated by the larger spread in their posterior belief distribution. This suggests their difficulty is in the updating rather than the formation of prior beliefs.

**Table 6:** Inconsistent vs. other updaters

	Min.	1st Qu.	Median	Mean	3rd Qu.	Max.
*Prior beliefs*						
Inconsistent updaters	0.00	0.04	0.25	0.32	0.50	0.99
Other updaters	0.00	0.10	0.25	0.31	0.50	0.95
*Posterior beliefs*						
Inconsistent updaters	0.00	0.04	0.26	0.32	0.51	0.93
Other updaters	0.01	0.10	0.16	0.21	0.26	0.92

## Discussion

Providing relevant and accurate information to the public is a central aspect of information-based health policies, but it does not suffice for the welfare assessment of such policies. Policy makers also need to know how (if at all) people respond to the provided information. Below, we discuss the findings of our study both on the individual and the aggregate level of belief revisions.

### Individual response to information

The key questions related to the efficacy of information-based health policies are who is going to respond to the information provided and by how much (Magat and Viscusi 1992; Viscusi 1998). We have examined patterns of belief revision including apparent heterogeneities in information take up. Surprisingly little of this heterogeneity is explained by the precautionary effort subjects made to reduce the risk of contracting a foodborne illness. Rather, we find that both prior and posterior risk beliefs increased with higher consumption of fish and concerns about seafood safety. The absence of a relationship between precautionary behavior and risk updating is consistent with the risk-as-feelings hypothesis (Loewenstein et al. 2001), which postulates that responses to risk information result in part from feelings such as worry, fear, dread, or anxiety that arise at the time of belief updating. These feelings may exert a negative influence on the cognitive evaluation of risk-related information, suppressing consideration of objectively risk-increasing or risk-reducing factors.

Recent research by Peters et al. (2006) finds that people differ in the degree to which they process risks cognitively versus affectively. In particular, their research indicates that highly numerate people draw more meaning from probabilities, frequencies, and other numerical comparisons than the less numerate do. In consequence, numerical risk information provides less meaning to individuals with lower numerical skills. Moreover, Peters and colleagues found that less numerate individuals are more prone to respond to irrelevant information suggesting that numerical information may even distort belief revisions. Their findings on the ability to process numerical information are consistent with our results indicating that less educated, less numerate, and older subjects were much more likely to inconsistently update their risk beliefs.

### Aggregate response to information

Even at the aggregate level, some noteworthy observations can be made on the response to risk information. Unlike a vast number of psychological studies (see Harris and Hahn 2011), we do not find unrealistic optimism about future life events. On average, our subjects were pessimistic when they formed their prior risk beliefs (*E*(*p*) = 31*%*) and remained slightly pessimistic (*E* [*q*] = 23*%*) upon receiving information about the population average risk of contracting a fishborne illness and the possible means to control this risk. Since the sample was constructed to be representative of French fish consumers, we would expect $\mathsf {E}\left [q\right ]\thickapprox s$ if subjects were true Bayesians. A Mann-Whitney test clearly rejects the hypothesized equality.

As the belief revision protocol was embedded in a larger survey on fish consumption, there might have been a salience effect at play. Recent and unusual events are more memorable and people therefore tend to draw on them when reasoning about experiencing similar events in the future (Gilbert and Wilson 2007). Yet the relative nature of the belief revision task emphasized both the risk of contracting a foodborne illness from fish as well as from other foods, making the salience hypothesis less plausible. Another possible explanation for control pessimism at the aggregate level is alarmist reactions to risk information (Viscusi 1997): people focus on worst-case scenarios when they update risk beliefs. For instance, some subjects might have believed that eating sushi would drastically increase their risk compared to the average consumer. While this would justify higher prior risk beliefs, it is hard to reconcile with higher posterior risk once we control for precautionary behavior. Lastly, we note that the sample median posterior risk belief was equal to the population average risk (*M* [*q*] = 16*%*), suggesting that control pessimism might be driven by outliers.

To explore the last explanation in more detail we compared inconsistent to consistent updaters and found a strong effect of information among consistent updaters, but no coherent effect among inconsistent updaters. This suggests that, as a group, inconsistent updaters could not infer much from the risk-related information. Yet since inconsistent subjects were about twice as often control-pessimistic as they were control-optimistic, we can also reject that they just made random belief updates. What is it then that drives inconsistent belief revisions? The results obtained from the mixture modeling suggest that inconsistent updaters tend to be older, less educated, and less likely to have passed the simple numeracy test than members of the other latent classes (albeit we do not find statistically significant differences between inconsistent updaters and information refuseniks). This highlights that specific groups within society are particularly prone to misunderstand health risk communications and that addressing these groups is indeed a challenge.

Heterogeneity in belief revision suggests that risk communication methods should be tailored to specific groups of recipients to obtain the optimal effects. Individuals who can process numerical risk information and update their prior beliefs in a consistent fashion may be well-served by providing such statistical information; in contrast, individuals who cannot consistently process such information may be better served by alternative risk communication, including perhaps messages that direct them to take specific actions (e.g., avoid raw fish, keep fish refrigerated until preparation, etc.). And if risk communication is crucial—e.g. in the case of a contagious disease—so called “fact boxes” (Gigerenzer and Kolpatzik 2017) may be more effective than the provision of raw statistical information. Another complication may arise from correlation neglect. In a belief updating task that was only slightly more complex than ours, Enke and Zimmermann (2018) found that many subjects failed to realize the correlation between the hints given to them. As a consequence, these subjects formulated the updating problem incorrectly even if they had the computational skills necessary to rationally update beliefs.

Individuals who fail to update their prior beliefs may already be well-informed about risks of consuming fish and other foods so that the information provided in the survey has negligible value; they may distrust the source of information and therefore ignore the information; or they may have simply failed to integrate the different pieces of information in a coherent way and, realizing this, may have decided to stick to their initial beliefs. Similar to correlation neglect, such information refusal can be seen as a mistake in conceptualizing the problem.

Although we have identified substantial heterogeneity in patterns of risk belief updating, we have only limited ability to predict how any individual will update. On average, individuals who are more numerate, highly-educated, and younger are better able to process numerical risk information and to update their prior beliefs in a consistent way, but these are weak predictors and conceal much variation. It may be useful in future work to identify more accurate methods for predicting how different individuals will respond to risk information, and what forms of communication are most effective for different individuals.

## Conclusion

The effectiveness of information-based policies is routinely predicted based on the assumption of Bayesian belief updating. Yet outcomes of information campaigns depend crucially on people’s actual response to the information disseminated, which in turn is contingent upon whether the information is taken up and processed in the expected way. In this paper we have explored the revision of risk beliefs in the realm of food safety. We find evidence for four patterns or strategies of updating beliefs about the risk of contracting a foodborne illness. The adoption of any of these strategies is not explained by differences in precautionary behavior, but is associated with factors determining the respondent’s age, numeracy, and educational attainment. Our results reinforce the common-sense notion that, because information campaigns are often targeted at demographic groups with limited numerical skills, policy makers need to make all efforts to communicate statistical information in the most accessible way possible—even at the cost of simplification—and consider how to best tailor informational content to people who differ in the type of assistance they need in making informed choices. Even if policy makers do so, one cannot expect that everyone will conceptualize the problem of merging statistical information with own behaviors and experiences correctly and this has to be taken into account when predicting the outcome of information-based policies.

## Appendix

In the empirical analysis, we employ the double-index beta regression model introduced by Ferrari and Cribari-Neto (2004) and refined by Smithson and Verkuilen (2006). This model consists of two index functions: one for the mean and one for the variance. To characterize the mean and precision functions as combinations of predictors, we parameterize the beta distribution as:

3 $$ f(y|\mu,\phi)=\frac{{\Gamma}(\phi)}{{\Gamma}(\mu\phi){\Gamma}((1-\mu)\phi)}y^{\mu\phi-1}(1-y)^{(1-\mu)\phi-1},\quad0<y<1, 0<\mu<1, \phi>0, $$

where Γ(⋅) denotes the gamma function. The beta-distributed response variable *y* has mean *E* [*y*] = *μ* and variance *V**a**r* [*y*] = *μ*(1 − *μ*)/(1 + *ϕ*), where *ϕ* is a precision parameter because, for *μ* fixed, the larger *ϕ* the smaller the variance. Assume now that the fractional responses of our *i* = 1, 2,...,*n* subjects are beta-distributed, i.e. $q_{i}\sim \mathcal {B}(\mu _{i},\phi _{i})$. The parameters *μ*_*i*_ and *ϕ*_*i*_ can be mapped onto the real line by appropriate link functions. We use the logit link for the mean function and the log link for the precision function, respectively:

4 $$\begin{array}{@{}rcl@{}} g(\mu_{i}) & = & \log(\mu_{i}/(1-\mu_{i}))=\beta^{\prime}\mathbf{X}_{i}\leftrightarrow\mu_{i}=\frac{\exp(\beta^{\prime}\mathbf{X}_{i})}{1+\exp(\beta^{\prime}\mathbf{X}_{i})}, \text{and} \end{array} $$

5 $$ h(\phi_{i})=\log(\phi_{i})=\zeta^{\prime}\mathbf{Z}_{i}\leftrightarrow\phi_{i}=\exp(\zeta^{\prime}\mathbf{Z}_{i}). $$

Here, **X**_*i*_ and **Z**_*i*_ are vectors of explanatory variables associated with the mean and precision parameter of the beta distribution, respectively. The corresponding coefficient vectors *β* and *ζ* are estimated by maximum likelihood techniques.

In order to implement the Bayesian learning model given by Eq. 2, **X**_*i*_ and **Z**_*i*_ need to be appropriately specified. Assuming substantial overlap between factors that influence the formation of the prior and the belief updating process, we stack all perceptual and behavioral factors (***A***_*i*_, ***B***_*i*_, ***C***_*i*_, and Δ**D**_*i*_) into the single vector **X**_*i*_. For the specification of the variance-determining vector **Z**_*i*_, we draw on Definition 2 and the concept of optimistic and pessimistic risk beliefs. As the population average risk of contracting a fishborne illness is 16*%*, it is likely that the updating behavior is more dispersed among baseline pessimists than among baseline optimists; similarly, the updating behavior is likely to be more dispersed among control pessimists than among control optimists. Estimating type-specific variances allows us to capture systematic differences in the updating behavior of optimists and pessimists.

So far, we have assumed that heterogeneity in belief updating can be explained by observable factors. However, latent traits may affect the formation and revision of risk beliefs. Finite mixture models have become a popular means to capture unobserved heterogeneity among decision makers (e.g., Houser et al. 2004; Andersen et al. 2008; Bruhin et al. 2010). The goal of mixture modeling is to estimate the set of parameters in *g*(⋅) and *h*(⋅) for each of *k* = 1, 2,…,*K* latent classes along with their membership function, so that the model predicts to which class each observation most likely belongs (McLachlan and Peel 2000).

Empirical applications of beta regression mixtures in the social sciences are still rare. Here, we follow studies by Smithson and Segale (2009) and Smithson et al. (2011) who estimate the beta regression mixture model:

6 $$ m(q|\mathbf{W},\mathbf{X},\mathbf{Z},\psi)=\sum\limits_{k = 1}^{K}\pi_{k}(\mathbf{W},\alpha_{k})f_{k}(q|\mu(\mathbf{X},\beta_{k}),\phi(\mathbf{Z},\zeta_{k})), $$

where the vector *ψ* = (*α*_1_,...,*α*_*K*_,*β*_1_,...,*β*_*K*_,*ζ*_1_,...,*ζ*_*K*_) collects the class-specific parameters of the discrete mixture density *m*(⋅); *f*_*k*_(⋅) is the density of the parameterized beta distribution that belongs to the *k* th latent class; and *π*_*k*_(⋅) is the corresponding membership function, which depends on a class-specific constant and, possibly, on concomitant variables summarized in the vector **W** (Dayton and Macready 1988). The membership function itself is conveniently modeled by the multinomial logit:

7 $$ \pi_{k}(\mathbf{W},\alpha_{k})=\frac{\exp(\alpha_{k}^{\prime}\mathbf{W})}{{\sum}_{u = 1}^{K}\exp(\alpha_{u}^{\prime}\mathbf{W})}, $$

whose identification requires normalizing the coefficient vector of class *c* to zero (i.e., *α*_*c*_ = 0).

The mixture model (6) has a non-trivial likelihood function, the maximization of which is cumbersome. We employ the expectation maximization (EM) algorithm (Dempster et al. 1977) as implemented in the *R* package ‘betareg’ (version 3.1-0). The EM algorithm is an iterative procedure for maximizing likelihood functions in a missing data setting. In the context of latent class mixture models, it is the class membership of the subjects that is unknown. The EM algorithm iterates between the E-step: evaluation of the expected complete-data log-likelihood given the observed data by fitting equation (7) to each observation; and the M-Step: maximization of the complete-data log-likelihood pertaining to Eq. 6, using previously derived individual posterior class probabilities as weights. In each iteration *j*, the E-Step calculates a Bayesian update of every subject’s posterior probability of belonging to class *k*:

8 $$ \tau_{ik}(q_{i}|\psi_{i}^{(j)})=\frac{\pi_{k}(\mathbf{W},\alpha_{k}^{(j)})f_{k}(q|\mu(\mathbf{X},\beta_{k}^{(j)}),\phi(\mathbf{Z},\zeta_{k}^{(j)}))}{{\sum}_{u = 1}^{K}\pi_{u}(\mathbf{W},\alpha_{u}^{(j)})f_{u}(q|\mu(\mathbf{X},\beta_{u}^{(j)}),\phi(\mathbf{Z},\zeta_{u}^{(j)}))}. $$

The iteration continues until the EM algorithm converges to a stationary point of the likelihood function (McLachlan and Peel 2000). In the empirical application, we set the convergence threshold to 1E-6 and repeated the estimation with 100 random starting values to reduce the risk of converging to a local maximum. As Bruhin et al. (2010) note, the final posterior probabilities of belonging to class *k* are a valuable result of the estimation process as this provides information on the sharpness of the classification. Moreover, the posterior probabilities enable us to estimate the average marginal effects that the concomitant variables reported in Table 4 had on the probability of belonging to each class by averaging over the individual marginal effects: $\frac {\partial \tau _{ik}^{(*)}}{\partial w_{ij}}=\tau _{ik}^{(*)}\left (\alpha _{jk}-\left ({\sum }_{u\neq k}\tau _{iu}^{(*)}\alpha _{ju}\right )\right )$. Table 7 displays the resulting average marginal effects.

**Table 7:** Average marginal effects of the concomitant variables reported in Table 4

	Inconsistent updaters	Reluctant updaters	Aggressive updaters	Information refuseniks
(*π*)_HEALTH STATUS	–0.8%	2.0%	0.1%	–1.3%
(*π*)_PREGNANCY	2.9%	3.4%	–5.5%	–0.8%
(*π*)_MALE	–3.1%	1.2%	3.7%	–1.9%
(*π*)_KIDS	8.4%	1.3%	–5.6%	–4.1%
(*π*)_AGE	0.5%	–0.3%	–0.2%	0.0%
(*π*)_INCOME	–3.5%	–0.6%	2.7%	1.4%
(*π*)_EDUCATION	–0.9%	–1.3%	1.9%	0.3%
(*π*)_NUMERACY	–18.1%	6.3%	18.7%	–6.8%
(*π*)_SUBJECTIVE RISK	–1.8%	13.3%	–12.5%	1.1%
(*π*)_SAFETY CONCERNS	7.0%	–4.1%	–3.2%	0.3%

## Footnotes

1 As people process information in accordance with their feelings about a particular source of risk (Loewenstein et al. 2001; Slovic et al. 2004), it is of little surprise that negative news attracts more attention. The emotional filtering is apparently reversed when subjects form and revise beliefs about desirable aspects of life (e.g., Eil and Rao 2011; Mobius et al. 2011).

2 Harris and Hahn (2011) warn that many of these studies might be methodologically flawed, however.

3 The Bayesian learning model presented in Eq. 1 can be derived by assuming beta-distributed beliefs. See Viscusi (1979) for a formal derivation in the context of insurance decisions with learning and Viscusi and O’Connor (1984) for the first empirical implementation in the context of chemical labeling.

4 Following Manski (2004, p.1343) we reminded subjects that “the percent chance must be a number from 0 to 100. Numbers like 2 or 5 percent may be ‘almost no chance’, 20 percent or so may mean ‘not much chance’, a 45 or 55 percent chance may be a ‘pretty even chance’, 80 percent or so may mean a ‘very good chance’, and a 95 or 98 percent chance may be ‘almost certain’.”

5 Epidemiological data for France (Vaillant et al. 2005) suggest that about 16*%* of all foodborne illnesses can be causally linked to fish. We also informed subjects that the average French consumer eats fish three times per week (based on information from the French Ministry of Agriculture, Food and Forestry, www.franceagrimer.fr); older people are more likely to contract a foodborne illness than younger; the majority of French consumers do not eat raw fish; and storing fresh fish for more than three days significantly increases the risk of contracting a foodborne illness.

6 Pretesting was done with administrative staffers of the Toulouse School of Economics and a convenience sample (*n* = 51) from the CSA panel. Quotas of the final sample were based on 2009 French census data (http://www.insee.fr/fr/recensement-2009.htm). The required minimum fish consumption imposed an additional eligibility constraint. The final response rate was 24%.

7 The regression equation counterpart of Eq. 2 thus is *q* = *β*_0_ + *β*_1_*p* + *𝜖*, where *𝜖* is a random error term.

8 To construct the counterfactual predictions, we replaced the observed values for the variables EAT FISH,RAW FISH,WASH HANDS,STORE FISH, andPREPARE FISH by sample maximum and minimum values while keeping all other variables fixed at sample means.

9 We keep predictors in the updating function *μ*_*k*_ and the membership function *π*_*k*_ separated as otherwise the identifying assumption of local independence might be violated (McLachlan and Peel 2000).

10 We settled on this model specification based on common goodness-of-fit measures for the mixture model *without* concomitant variables. A three-class mixture model with concomitant variables resulted in an even better fit, but the obtained classification is close to the one presented, the main difference being that information refuseniks and reluctant updaters are combined into a single class.

11 Coefficients of the concomitant variables need to be carefully interpreted to avoid confusion between relative effects (e.g. ‘how much more likely is a subject to have flunked the numeracy test when classified as an inconsistent updater?’) and absolute effects (e.g. ‘how much more likely is a subject to be classified as inconsistent when they flunked the numeracy test?’). Answering the latter question requires calculating marginal effects.
